# Refractive changes and visual quality in patients with corneal edema after cataract surgery

**DOI:** 10.1186/s12886-022-02452-5

**Published:** 2022-06-02

**Authors:** Mª Amparo Díez-Ajenjo, Mª José Luque-Cobija, Cristina Peris-Martínez, Susana Ortí-Navarro, Mª Carmen García-Domene

**Affiliations:** 1 FISABIO Oftalmología Médica (FOM), Bifurcación Pío Baroja-General Avilés, s/n. E46015 Valencia, Spain; 2 Optics, Optometry and Visual Sciences Department, Physics Faculty, University of Valencia, Av Dr. Moliner, 50, E 46100 Burjassot Valencia, Spain; 3 Cátedra Alcon-FISABIO-UVEG, Valencia, Spain; 4 Surgery Department, University of Valencia, Av Blasco Ibáñez 15, 46010 Valencia, Spain

**Keywords:** Cataract surgery, Contrast sensitivity function, Corneal edema, Clinical refraction, Visual acuity

## Abstract

**Background:**

To assess visual quality and stabilization of refractive changes in corneal edema patients after cataract surgery, using visual acuity (VA) and contrast sensitivity measurements.

**Methods:**

Sixty-one eyes were analysed, twenty-three with and thirty-eight without corneal edema. Uncorrected and corrected distance VA (UDVA and CDVA) were determined with an EDTRS chart, the contrast sensitivity function (CSF) under photopic and mesopic illumination conditions with a CVS-1000e chart, clinical refraction, and corneal topography. Measurements were taken preoperatively, 1–2 days, 1 and 3-months after surgery. Clinical refraction was converted to vector notation (M, J_0_, J_45_) and SPSS v26.0 was used for data analysis.

**Results:**

An improvement of VA was observed through the postoperative period; changes between visits were significant for CDVA in both groups and for UDVA in the edema sample. Significant astigmatic changes (J_0_,J_45_) between visits were not observed, but M values showed a hyperopic tendency in the edema group and a myopic shift in the control group that did not change between visits, with statistically significant differences between groups. Controls had significantly better contrast sensitivity at high spatial frequencies. Under mesopic conditions, global contrast sensitivity losses were observed in the edema group, which improved between visits in the middle frequency range.

**Conclusion:**

Corneal edema patients had a significant reduction of CDVA, and frequency-selective sensitivity losses that evidence a visual quality loss. Clinical refraction may improve visual quality, but in edema patients these losses are related to corneal changes, which did not change at three months after surgery.

## Background

New phacoemulsification surgery techniques have resulted in a trend towards improved visual acuity (VA) with minimum invasiveness and optimal safety. However, corneal edema delays early vision improvement after surgery [1–3]. The transient post-operative corneal swelling that commonly occurs after phacoemulsification is probably the result of damage to endothelial cells during surgery [4, 5] due to mechanical or chemical injury,—including larger corneal incision sizes [6, 7], direct mechanical trauma, the effect of ultrasound energy and the irrigating solution [2]-, subsequent inflammation or infection, pre-existing endothelial disease [4, 5, 8–10] or systemic diseases, such as diabetes [11]. Usually, corneal edema is reversible, but recovery times as different as one week [7, 12, 13], four weeks [12, 14, 15], 60 days [15, 16], six months [5, 17] or even one year after surgery [18] have been reported.

Theoretically, morphological corneal changes due to edema, such as keratometric changes or an increase of corneal thickness, could lead to a change in refractive corneal power. Possible corneal refraction index changes due to the hydration of certain components of the corneal tissues may also contribute to clinical refraction changes [19]. However, the extant bibliography does not offer evidence about which mechanisms would be responsible for refraction changes, nor show whether these changes are transient or permanent. This fact could be relevant to choose the ideal moment to prescribe clinical refraction.

There is not a single reference method to assess corneal edema after surgery. Slit-lamp examination is extensively used [20] although it is subjective, depends on the practitioner expertise and may pass as normal corneas with subclinical edema. To overcome these problems, different objective methods have been proposed, including specular microscopy and in vivo confocal microscopy for endothelial morphology [8, 21], pachymetry for central corneal thickness (CCT) [8, 21, 22], ocular coherence tomography (OCT) [6, 12], and densitometry using a rotating Scheimpflug camera [4]. However, The focus of this literature is on the changes in the cornea, and an analysis of the visual effects of corneal edema and data showing whether these visual effects are transient or permanent is missing. This paper aims to study the effects of corneal edema in optical and visual quality in patients implanted with an intraocular lens after cataract surgery.

## Methods

### Study design and participants

This is a retrospective study. Data was obtained from a previous prospective study about visual quality with intraocular lenses (IOL) that revealed differences in refraction between patients with and without corneal edema after cataract surgery, suggesting the need for further analysis.

The study adheres to the tenets of the Declaration of Helsinki for Research Involving Human Subjects and was approved by the Institutional Review Board and by the Ethics Committee of FISABIO Medical Ophthalmology. All patients were informed before entering the study and signed a written informed consent form for the prospective study, expressly consenting to the use of patient data for research.

Sixty-one eyes of sixty-one cataract surgery patients, 37 males and 24 females with mean age 73 ± 7 years, and implanted with the same monofocal IOL, were evaluated. The inclusion criterion was presence of senile cataracts Lens Opacities Classification System III (LOCS III) grade 2 or higher. Exclusion criteria were age-related macular degeneration or any ocular pathology which could distort the results, previous ocular surgery, ocular surface diseases, abnormalities in the endothelial quality or cell count lower than 2000 cells/mm^2^, ocular treatment of any type 1-month before surgery, medication that could cause drowsiness or a history of drug or alcohol addiction.

Patients were excluded from the study database if at any time an anomaly in the tests indicated that they might have been suffering from pathologies that would mask the results.

All the patients were examined four times: before surgery, 1–2 days, one month and three months after surgery. Before surgery, the patient’s corneal topography was measured with a Pentacam® HD device (Oculus, Wetzlar, Germany) and their endothelial cell count was determined by Topcon SP-2000P specular microscopy (Topcon America Corp, Paramus, NJ). An experienced ophthalmologist analysed the patient’s corneal status by slit-lamp microscopy, to exclude patients with corneal dystrophies.

At the 1–2 days’ visit, an experienced ophthalmologist evaluated corneal status with non-contact specular microscopy, to estimate CCT, endothelial cell density, morphology, and postsurgical corneal edema. In this visit, uncorrected distance VA was evaluated with and without a pinhole (UDVA_p_ and UDVA respectively). This is not the visual quality analysis protocol used in subsequent visits, because corneal stability is not reached yet, and it is very difficult to obtain a reliable clinical refraction [23]. Patients were included in the edema group when the ophthalmologist observed slightly lustreless or hazy corneas, increased corneal thickness on slit lamp biomicroscopy in comparison to preoperative values, and the pinhole did not improve VA.

Twenty-six eyes developed post-surgery corneal edema. To avoid duplicities, only one eye per patient was included, resulting in an edema group with twenty-three eyes and a control group with thirty-eight eyes.

In the first, third and fourth visits, the patient’s UDVA and corrected distance VA (CDVA) were measured with an EDTRS chart. The contrast sensitivity function (CSF) was determined with a CVS-1000e chart under photopic (85 cd/m^2^) and mesopic illumination conditions (4 cd/m^2^) after checking the illumination level with a HD 9221 digital photo-radiometer. The corneal topography was obtained at the third and fourth visits (one and three months after surgery, respectively).

### Surgical technique

Patients were implanted an AcrySof® IQ SN60FW lens (Alcon Laboratories, Inc. Fort Worth Texas, USA) using topical anaesthesia. In all cases, centred circular capsulorhexis not greater than 5 mm was performed. Standard phacoemulsification was performed using the Infinity System platform (Alcon Laboratories, Inc. Fort Worth Texas, USA). After irrigation and aspiration of the cortex, the IOL was implanted in the capsular bag using the Monarch II injector, through a corneal incision of approximately 2.75 mm. The same experienced surgeon (CP) performed all cataract surgeries.

All patients received the same postoperative antibiotic and topical corticoid treatment, consisting in Tobramicine and Dexamethasone (Tobradex Ophthalmic Suspension, Alcon Cusí, Barcelona, Spain) for four weeks at a dosage that was gradually decreased.

### Statistical analysis

Data were analysed using SPSS v26.0. Normal distribution of variables was assessed using the Kolmogorov–Smirnov test. Repeated-measures analysis of variance (ANOVA) was carried out to gauge any statistically significant difference within the results obtained in the different visits (1 day, 1 and 3-months after surgery). Post hoc multiple comparison testing was performed using the Bonferroni test. Student’s t-test was also used to check for statistically significant differences. A paired samples test was used to assess changes in time an unpaired sample test to compare between groups. Differences were considered to be statistically significant when *p* < 0.05.

## Results

Mean age was 74 ± 8 years for the edema group and 72 ± 8 years for the control group (*p* = 0.13). Preoperative CDVA was 0.32 ± 0.20 logMAR (21/40 Snellen equivalent) for the edema group and 0.33 ± 0.23 logMAR (22/40 Snellen equivalent) for the control group (*p* = 0.82). Preoperative endothelial cell count was 2432 ± 450 cells/mm^2^ for the control group and 2546 ± 595 cells/mm^2^ for the edema group, without statistically significant differences (*p* = 0.43). Mean preoperative corneal thickness was 575.0 ± 35.0 microns for edema patients and 557.0 ± 29.0 microns for the control group (*p* = 0.06).

**Fig. 1:**
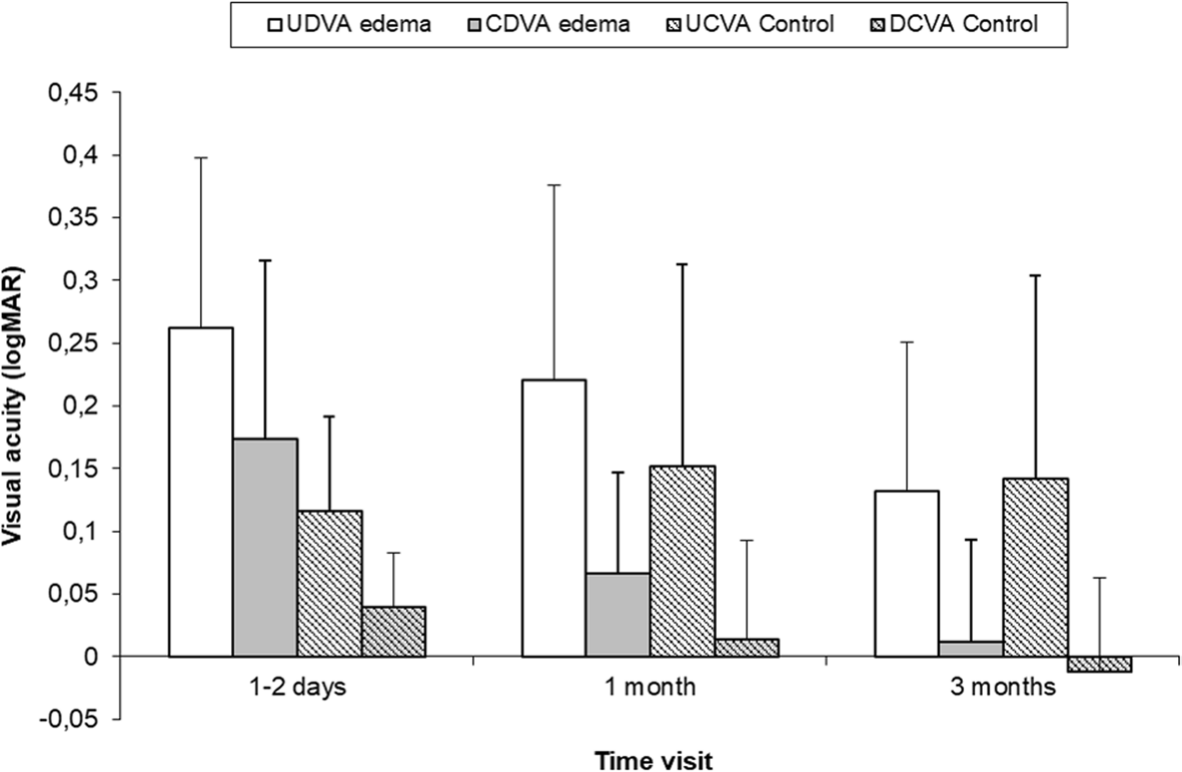
UDVA (white) and CDVA (grey) for controls (striped bars) and edema (uniform bars) at each visit. At the 1–2 days postoperative visit, UDVA_p_ instead of CDVA is shown

Figure 1 shows UDVA and CDVA for edema patients and controls at the four post-surgery visits. Optical compensation significantly improves VA, except for the edema group at the 1–2 days visit (*p* = 0.18). A statistically significant improvement in VA between visits is also observed. The control group shows worse UDVA after the 1–2 days visit, but this decrease is not statistically significant (*p* = 0.73 for the post-surgery and 1-month visits, *p* = 0.76 for the 1-month and 3-months visits, and *p* = 0.27 for the post-surgery and 3-months visits). A slight improvement in CDVA can be observed for controls, but the only significant change appears between the 1-month and the 3-months visit (*p* = 0.008).

The control group exhibits better VA than edema patients, but the difference between groups is significant only at the 1–2 days post-surgery visit for UDVA and CDVA (*p* < 0.001 and *p* = 0.006, respectively) and for CDVA at the 1-month visit (*p* = 0.011).

**Fig. 2:**
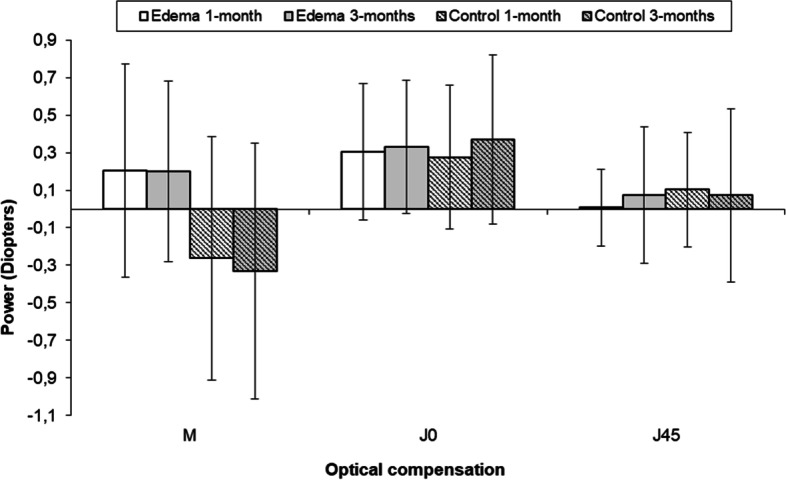
M, J_0_ and J_45_ values for controls (striped bars) and edema (solid bars) s at the 1-month (white bars) and the 3-months (grey bars) visits

For the analysis of the clinical refraction, power vector components- spherical equivalents M, ortho-astigmatism J_0_ oblique astigmatism, J_45_ were computed from the spherocylindrical refraction [24]. The values obtained at the 1-month and the 3-months visits are shown in Fig. 2. The astigmatic components J_0_ and J_45_ do not significantly change between visits in either group. Changes in the spherical equivalent M show a slight hyperopic tendency in the edema group and a myopic shift in controls at the 1 and 3-months visits, with significant differences between groups at both visits (*p* = 0.02 and *p* = 0.006 for the 1-month and 3-months visits, respectively). The slight differences between visits for a given group, however, are not statistically significant.

**Fig. 3:**
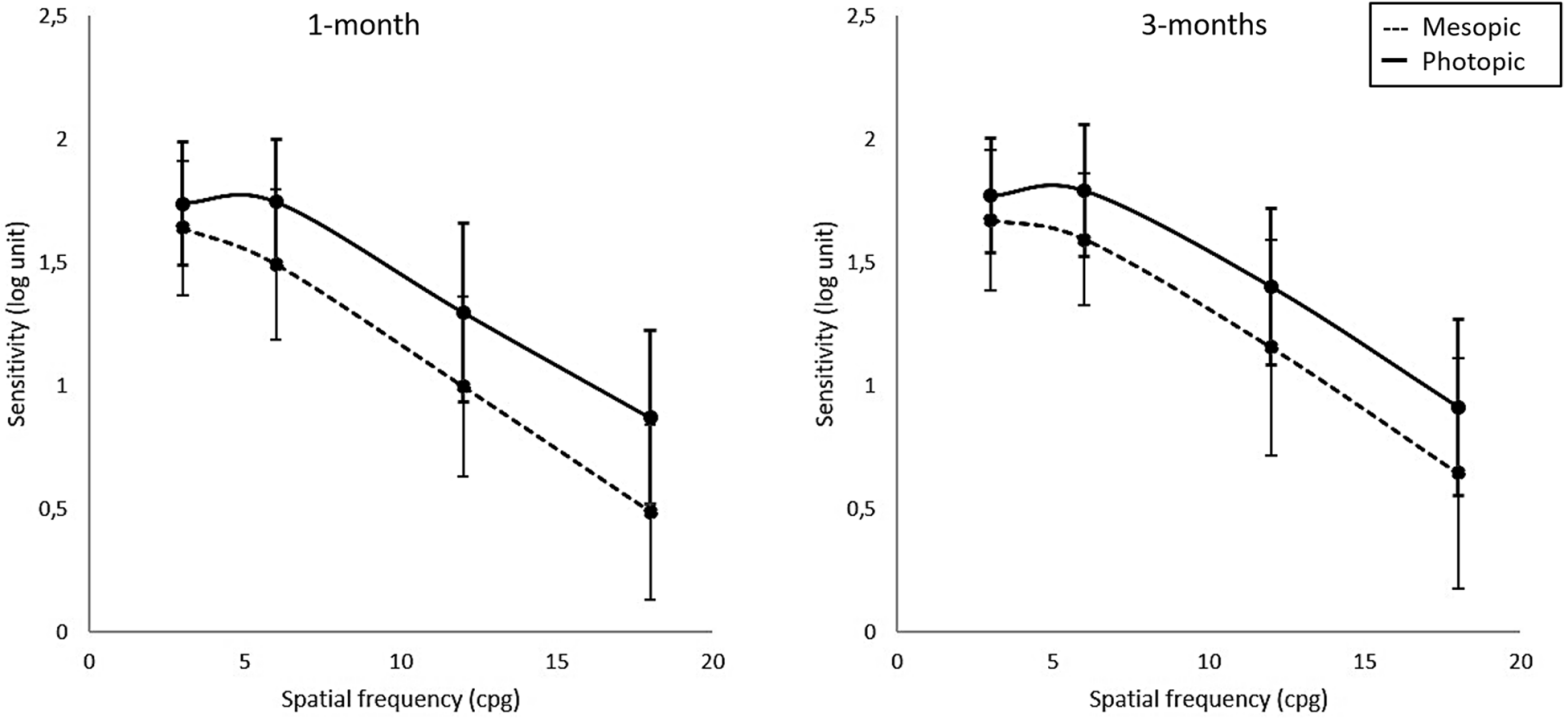
Contrast sensitivity function for controls at the 1-month (left) and 3-months (right) visits. The continuous line corresponds to photopic illumination conditions, and the dashed line to mesopic illumination conditions

Figure 3 shows the average CSFs for edema patients. Photopic and mesopic CSFs improved from the 1-month to the 3-months visit, but these changes were significant only for 6, 12 and 18 cpd under mesopic conditions (*p* = 0.002, *p* = 0.011 and *p* = 0.021 respectively). Differences between photopic and mesopic CSFs were statistically significant for all frequencies at both visits.

**Fig. 4:**
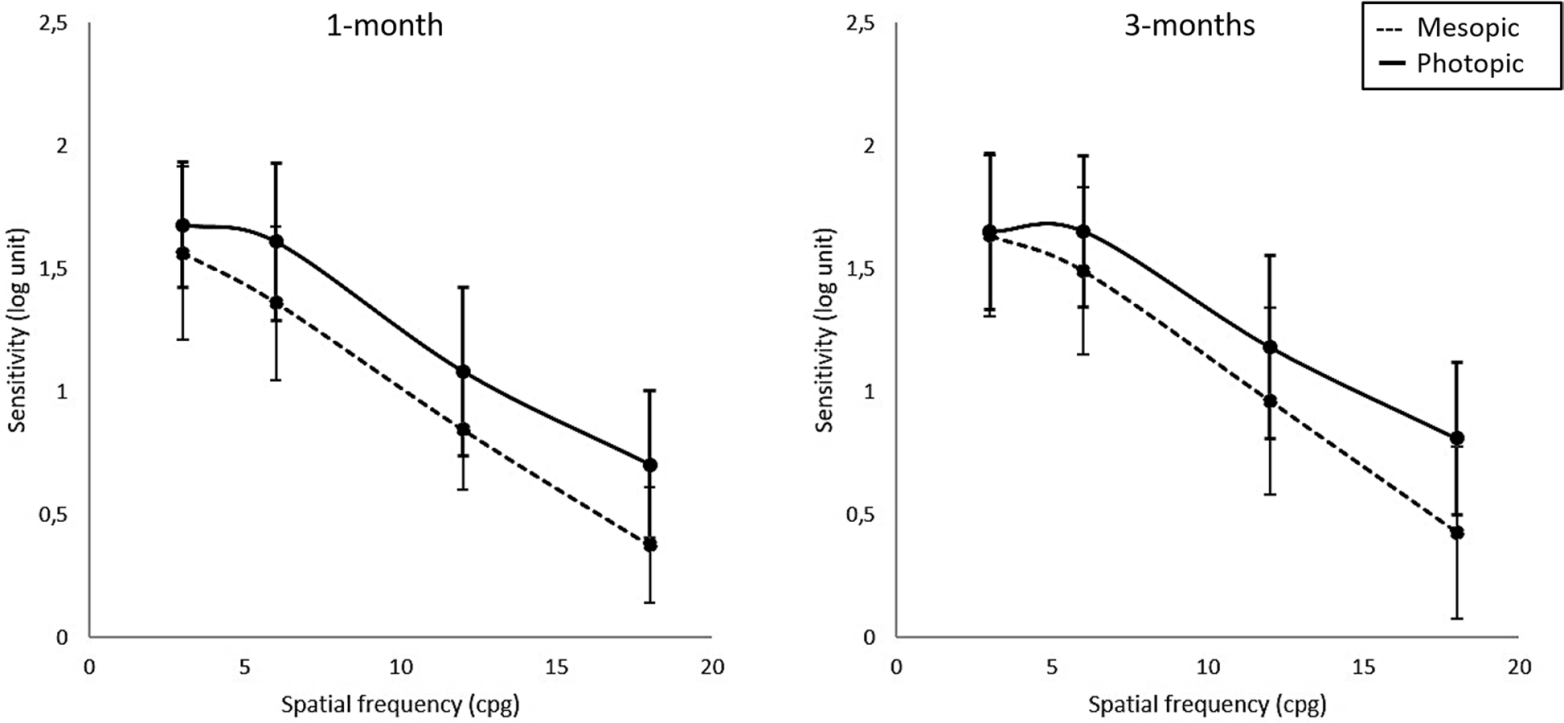
Contrast sensitivity function for the edema group at the 1-month (left) and 3-months (right) visits. Continuous line: photopic illumination conditions. Dashed line: mesopic illumination conditions

Figure 4 shows the CSF obtained for edema patients. Between the 1-month and 3-month visits, a trend to improved contrast sensitivity is detected, although changes are not statistical significant. However, results suggest that this improvement could be greater under mesopic conditions: though in the 1-month visit mesopic sensitivity is significantly lower than photopic sensitivity at all frequencies, as expected, at the 3-month visit significant differences only occur at frequencies above 1 cpd.

**Fig. 5:**
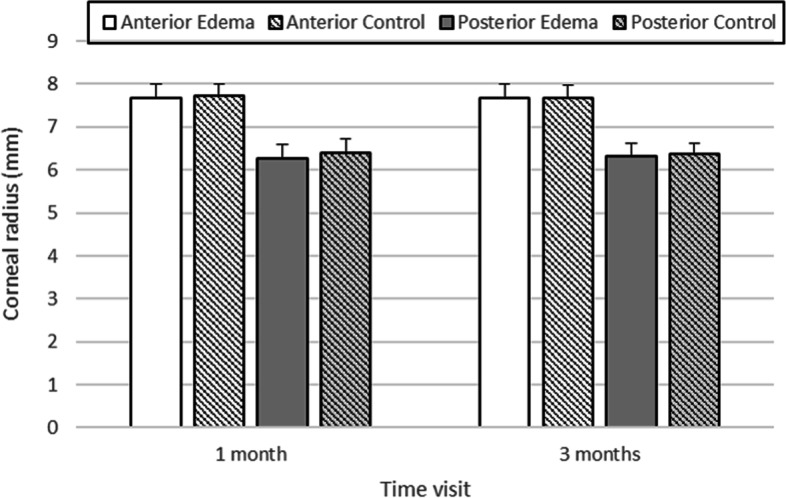
Anterior (white bars) and posterior (grey bars) corneal radius for controls (stiped bars) and edema patients (solid bars)

CSFs were better in controls than in edema patients for all visits and under both illumination conditions. However, these differences were significant only under photopic conditions and at high spatial frequencies at the 1-month visit (*p* = 0.014 for 12 cpd and *p* = 0.03 for 18 cpd) and for 12 cpd at the 3-months visit (*p* = 0.03) for mesopic conditions. The changes in anterior and posterior mean corneal radius changes at each post-surgery visit for the edema and control samples are shown in Fig. 5. The differences observed between both visits for each group and between both groups are not statistically significant. CCT was 579 ± 47 µm and 576 ± 41 µm in the edema groups in the 1-month and 3-month visit, respectively, while for controls the values were 555 ± 26 µm and 558 ± 28 µm. Although the edema group had, in average, thicker corneas than controls, these differences were not significant (*p* = 0.06 for the 1-moth visit and *p* = 0.09 for the 3-month visit).

## Discussion

It is difficult to find in the literature studies about visual quality of patients with corneal edema after cataract surgery. There is abundant literature concerning clinical refraction stabilization of patients without post-surgical complications [23, 25], but the situation changes in corneal edema patients. This may be because poor visual quality in these patients is assumed, and because visual quality depends on the severity of corneal edema, introducing large variability in the samples. Morphological changes and loss of transparency, which can also be maintained in time, impair the visual quality of these patients. If corneal edema can take as long as one year after cataract surgery to disappear [18], the visual quality of these patients must be assessed and measures taken to improve it, when possible. Delays in prescribing spectacles are inconvenient for patients and may negatively affect their quality of life during the postoperative period, as can be concluded from our results.

Corneal edema produced after cataract surgery changes certain corneal physiological parameters. CCT increases due to the inflammation produced [26]. In our sample, CCTs were below 650 μm and the edematous corneas were not too thick and opaque. Therefore, overestimation in the Pentacam measurements, due to problems with the device’s light wavelength [27], is not expected. Although the refractive changes observed could reasonably be linked to changes in corneal radius, our measurements did not reveal corneal radius differences between controls and edema patients. This behaviour agrees with previous results with artificial corneal edema [28, 29], provoked by exposing the cornea to an anoxic environment for 2 h, using a nitrogen chamber goggle. Although in our study the causes of the edema are different, the same corneal effects can be assumed.

The hydration of certain corneal tissues is altered in different degrees and their refractive indices changes accordingly [30]. Our refraction results suggest a change in corneal refraction index with edema, dependent on the severity of the hydration, because refraction changes cannot be explained by CCT changes only. Meek et al. [30] measured and modelled only the stroma’s refraction index. Their model predicts that the refractive index of the swollen stroma depends on only two parameters in the physiological stroma, the refractive index of the cornea before the edema, and the hydration produced. This dependence was hyperbolic. It is true that the behaviour of the stroma is not necessarily extensible to the whole cornea, but since it constitutes approximately 90% of the cornea, the variation of its refraction index can be a good approximation of the total corneal changes.

All these morphological changes affect visual refraction. Analysing refractive changes, it was found that refractive values did not change between visits. J_0_ was greater than J_45_ in control and edema patients, so surgery produces regular astigmatisms in both groups. Astigmatism did not significantly change between visits, in agreement with the absence of significant changes in corneal radii during the three months evaluated.

The spherical equivalent M showed a clear hyperopic shift due to edema. This fact suggests a decrease of corneal power that, with our data, only can be explained by an increase of CCT and a decrease of corneal refraction index [30]. It seems, therefore, reasonable to expect that this hyperopic shift would disappear when hydration values return to normal. Studies with additional post-surgical visits beyond our 2-month interval are necessary to confirm this hypothesis.

Regarding visual quality, CDVA losses were observed in edema patients. It seems obvious that the observed refractive changes produce poor visual quality. If refractive errors are not compensated, the visual quality of edema patients will be compromised, but, on the other hand, clinical refraction may change in a short time. However, an improvement was observed between visits without a change in clinical refraction change, which might be due to an improvement in corneal transparency. The contrast sensitivity function exhibited an analogous behaviour. Patients showed a sensitivity decrease at high spatial frequencies, consistent with the impairment in CDVA. The sensitivity loss was greater under mesopic conditions, which could be explained by light scattering due to transparency loss in edematous corneas.

Our study has limitations. It cannot be affirm whether clinical refraction differences in edema patients will be permanent or not. According to our results, it can be predicted that these changes will disappear when the CCT and the corneal refraction index return to normal values. The time required for this recovery cannot be accurately predicted from our data. Moreover, slit lamp examination to classify edema patients is a subjective method and patients with slit-lamp undetectable edema may be included in the control group. Although the same experienced ophthalmologist classified corneal edema, as Ishikawa et al. [4] commented, there are incipient corneal edemas that could be detected by Scheimplflug densitometry, buy could be undetectable by slit lamp examination. If this case occurred, at least it can be confirmed that these patients did not have visual symptoms, since they had a good VA.

Although the patients included in our study had not severe corneal edemas, a classification of edemas according to severity would be necessary for more accurate understanding of the visual impairment caused by this condition, because corneal physiological changes depend on the degree of corneal hydration. In our case, this classification seems not to be determinant, since edema patients and controls show the same dispersion in the results.

## Conclusions

Our study demonstrates that corneal changes due to edema affects clinical refraction and these changes persist three months after surgery, probably because the corneal refraction index and CCT are still altered. These corneal changes affect visual quality, especially under mesopic conditions, due to loss of transparency. It is necessary to decide for each individual patient whether to improve or not his or her visual quality using an optical correction that will probably change after a brief period.

## Data Availability

The datasets used and/or analysed during the current study available from the corresponding author on reasonable request.

## Abbreviations

ANOVA: Analysis of variance
CCT: Central corneal thickness
CDVA: Corrected distance visual acuity
CDVA: Corrected distance visual acuity
CSF: Contrast sensitivity function
IOL: Intraocular lens
OCT: Ocular coherence tomography
UDVA: Uncorrected distance visual acuity
UDVA_p_: Uncorrected distance visual acuity with a pinhole
VA: Visual acuity
